# RNA 3D Structure Prediction Using Coarse-Grained Models

**DOI:** 10.3389/fmolb.2021.720937

**Published:** 2021-07-02

**Authors:** Jun Li, Shi-Jie Chen

**Affiliations:** Departments of Physics and Biochemistry, and Institute of Data Science and Informatics, University of Missouri, Columbia, MO, United States

**Keywords:** RNA, structure prediction, coarse-grained, molecular dynamics, monte carlo, statistical potential, all-atom force field

## Abstract

The three-dimensional (3D) structures of Ribonucleic acid (RNA) molecules are essential to understanding their various and important biological functions. However, experimental determination of the atomic structures is laborious and technically difficult. The large gap between the number of sequences and the experimentally determined structures enables the thriving development of computational approaches to modeling RNAs. However, computational methods based on all-atom simulations are intractable for large RNA systems, which demand long time simulations. Facing such a challenge, many coarse-grained (CG) models have been developed. Here, we provide a review of CG models for modeling RNA 3D structures, compare the performance of the different models, and offer insights into potential future developments.

## 1 Introduction

Ribonucleic acid (RNA) is an unbranched polymeric macromolecule undertaking crucial and various biological functions, such as carrying genetic information, directing protein synthesis, regulating gene expression, catalyzing biological reactions (Gesteland et al., 2005; Cech and Steitz, 2014; Morris and Mattick, 2014; Mortimer et al., 2014). Exogenous RNAs can also exert deleterious effects on human health. For example, SARS-CoV-2, an RNA virus, caused the serious respiratory disease COVID-19 in the current pandemic (Tu et al., 2020; Oran and Topol, 2020). Moreover, recently developed mRNA vaccines suggest that RNAs can be used as an antiviral therapeutic agents against COVID-19 by making antibodies through human immune response (Amanat and Krammer, 2020; Krammer, 2020; Moore and Offit, 2021). RNA functions are tied to their 3D structures. For example, the formation of pseudoknot structure in SARS-CoV-2 frameshift stimulating element is required for ribosomal frameshifting which can lead to the production of alternative proteins from the same genomic region (Kelly et al., 2020). Therefore, detailed RNA 3D structures are important for understanding their functions and for drug design. To determine high-resolution 3D structures, X-ray crystallography and nuclear magnetic resonance (NMR) spectroscopy experiments are conventionally performed, but it is laborious and tough to solve structures by these methods. Meanwhile, numerous RNA sequences have been obtained but their 3D structures remain unknown. Consequently, the huge gap between the number of sequences and the known structures stimulates the development of computational 3D structure prediction methods over the past decades (Miao and Westhof, 2017). In terms of the resolution of the RNA structural model, these methods can be classified into two categories, namely all-atom (AA) and coarse-grained (CG) approaches. For AA approaches, 20–23 heavy atoms and 10–11 hydrogen atoms are considered for each nucleotide, while for some CG approaches, several atoms are grouped together into a bead and only several beads are used to model a nucleotide, and for some other CG approaches, even more coarse-grained representations are used by treating helix and loop structures as vertices and edges. For *de novo* modeling methods without using solved homologous structures, sampling in the energy landscape is often required and the lowest free energy structure is searched for (Anfinsen, 1973). Compared with AA methods, the energy landscape in CG models is smoother, enabling faster arrival at the global energy minima, and the computation should be more rapid due to fewer atoms. Next we will introduce a dozen of CG approaches to modeling RNA 3D structures.

## 2 Coarse-Grained Models for Ribonucleic acid 3D Structure Modeling

**FIGURE 1:**
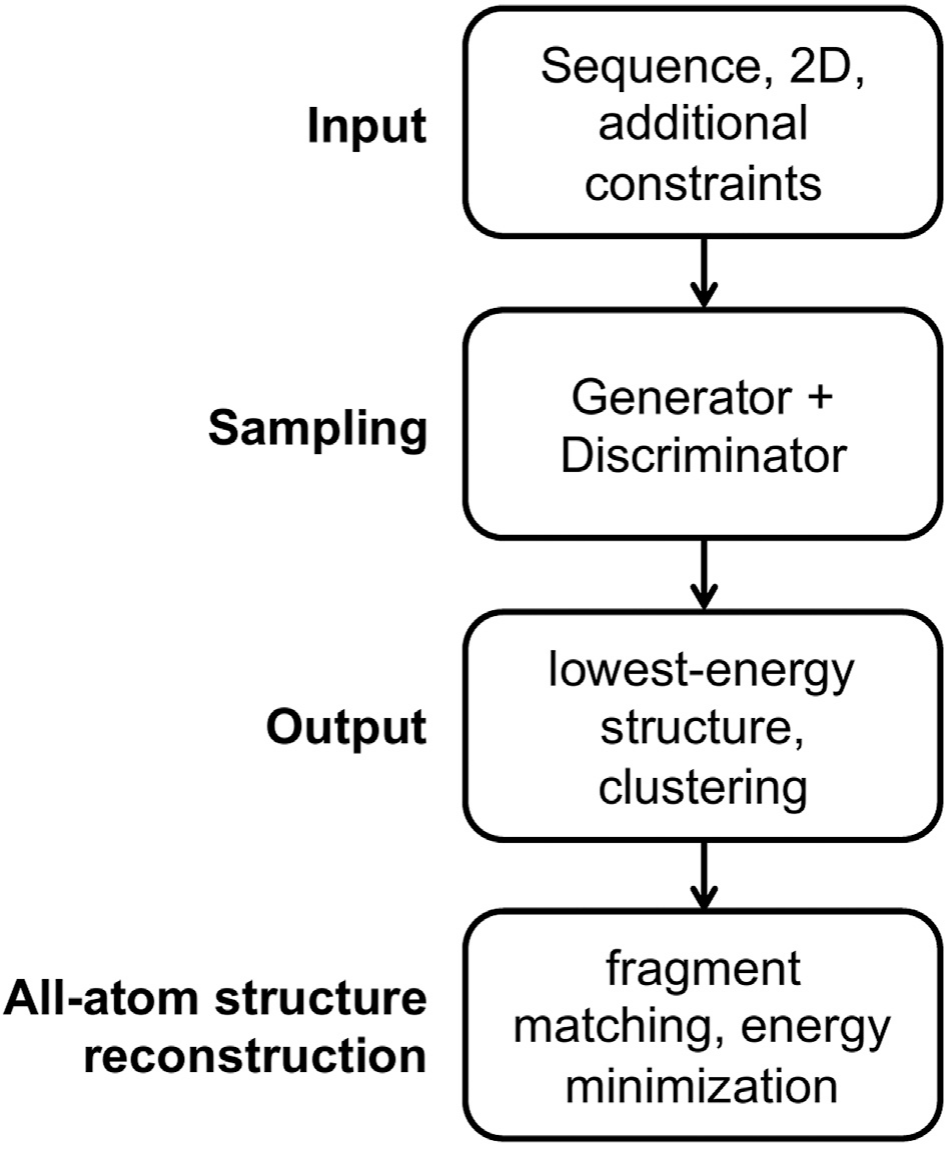
The general workflow for predicting RNA 3D structures using CG models.

The general workflow for predicting RNA 3D structures using CG models is shown in Figure 1. There are four parts in the workflow, namely input, sampling, output and all-atom structure reconstruction. We will go through them next.

**Input** For the input part, an RNA sequence is usually required, and a secondary structure is essential for some models and is optional for others. Additional constraints are applicable for some models, such as experimental information, distance between specified atom pairs, initial 3D structures, and templates for motifs.

**Sampling** For the sampling part, a conformational generator for producing candidate structures and a conformational discriminator for assessing structures are essential. A generator is commonly implemented in a Monte Carlo (MC) or molecular dynamics (MD) simulation framework, and many variants of MC and MD engines are utilized to accelerate conformational sampling. Generally, a discriminator is called scoring function, (potential) energy function, or force field in structure prediction field. There are three main forms of energy functions used in the CG models. The first type of CG energy function mimics the all-atom force field including the bonded terms of bond lengths, angles and torsions, and the non-bonded terms of Van der Waals and electrostatic energies, as shown in Eq. 1 for the AMBER force field (Wang et al., 2004) where harmonic potentials are used for bonds and angles, a cosine form potential is used for torsions, the Lennard-Jones potential is used for Van der Waals interactions, and the Coulomb interaction energy is used for electrostatic interactions.

$$\begin{array}{l} E_{\text{AMBER}}=\underset{\text{bonds}}{\sum }K_{r}{\left(r-r_{\text{eq}}\right)}^{2}+\underset{\text{angles}}{\sum }K_{\theta }{\left(\theta -\theta_{\text{eq}}\right)}^{2}+\underset{\text{torsions}}{\sum }\frac{V_{n}}{2}\left[1+\text{cos}\left(n\phi -\gamma \right)\right]\\ +\underset{\begin{array}{c} i<j\\ \text{Van}\;\text{der}\;\text{Waals} \end{array}}{\sum }\left(\frac{A_{ij}}{R_{ij}^{12}}-\frac{B_{ij}}{R_{ij}^{6}}\right)+\underset{\begin{array}{c} i<j\\ \text{electrostatic} \end{array}}{\sum }\left(\frac{q_{i}q_{j}}{\epsilon R_{ij}}\right) \end{array}$$ (1)

The second type of CG energy function is the potential of mean force (PMF) (Sippl, 1990) using the inverse Boltzmann formula as shown in Eq. 2, where E(x) is the PMF for a structural variable *x*, $k_{\text{B}}$ is the Boltzmann constant, *T* is the temperature, $p_{\text{obs}}\left(x\right)$ is the probability distribution for *x* observed in the experimental structures, and $p_{\text{ref}}\left(x\right)$ is the probability distribution for *x* in the reference state. The common structural variable *x* is the nucleotide- or atom-dependent interatomic distance, angle, torsion or the combination of them. We also call this potential statistical potential or knowledge-based potential (Capriotti et al., 2011; Wang et al., 2015; Tan et al., 2019).

$$E\left(x\right)=-k_{\text{B}}T\text{ln}\;\;\left[p_{\text{obs}}\left(x\right)/p_{\text{ref}}\left(x\right)\right]$$ (2)

The third type of CG energy function is the hybrid of the former two energies. For example, for the bonded energies, the harmonic, Gaussian or other energy functions are used mimicking the all-atom force field, while for the non-bonded energies, the PMF energy is used to account for base pairing and base stacking interactions. The parameters in the bonded energy functions are usually fitted to the statistical potentials derived from the experimental structures (Tan et al., 2006; Jonikas et al., 2009b; Xia et al., 2013). In addition to the above three main forms of energy functions, some models use the sum and product of several harmonic potentials, Gaussian energy functions or other functions as a combined complex energy function (Šulc et al., 2014; Zhang et al., 2021). Some CG models can integrate additional experimental information into the energy functions by transforming the information into distance, angle, torsion and other constraints (Tan et al., 2006; Jonikas et al., 2009b; Xia et al., 2013; Krokhotin et al., 2015). Generally the conformational generator and discriminator are not independent. Ideally the discriminator would lead the generator towards the low-energy conformational space. However, the generator may be trapped in the local minima in the energy landscape, and advanced sampling algorithms may help the generator traverse these local minima efficiently.

**Output** For the output part, some models select the structure with the lowest energy as the predicted native structure, while other models first group the low-energy structures into several clusters, and choose the centroid structures in the clusters as the predicted structures. The predicted best centroid structure is from the largest cluster, which accounts implicitly for the entropy effect.

**All-atom structure reconstruction** All-atom structures are essential for studying ligand binding and drug design and for structure-based understanding of RNA functions. For the all-atom structure reconstruction part, the fragment matching algorithm (Jonikas et al., 2009a) is often used, followed by structure refinement to remove steric clashes and chain breaks.

### 2.1 Models

Twelve CG models are described next and summarized in Table 1. The models are sorted first by the CG level and then by the published time.

**TABLE 1:** Summary of CG models for RNA 3D structure modeling.

Model	Representation	Energy	Applicable constraints^a^	Sampling	Output	Availability
Graph	Helix-based	KB^2^	No	MC^7^ + SA^8^	AA	No
Ernwin	Helix-based	KB	No	MCMC^9^	CG^15^	Standalone + server
YUP	1 bead/nt^1^	KB	Footprinting, cross-link, etc.	MC + SA	CG	No
NAST	1 bead/nt	KB	Tertiary contact	MD^10^	CG/AA^16^	Standalone
iFoldRNA	3 beads/nt	KB + TD^3^	HRP^4^, NMR^5^	REDMD^11^	AA	Server
TOPRNA	3 beads/nt	KB	No	REMD^12^	CG	Standalone
IsRNA1	4–5 beads/nt	KB	No	REMD	AA	Standalone + sever
Ren’s	5 beads/nt	KB	NMR, SAXS^6^	MD/SA	AA	No
oxRNA	5 beads/nt	TD	No	MC + US^13^ + MD	CG	Standalone + server
SimRNA	5 beads/nt	KB	Distance	REMC^14^	AA	Standalone + server
SPQR	5 beads/nt	KB	No	MC + SA	AA	Standalone
HiRE-RNA	6–7 beads/nt	KB	No	REMD	AA	No

a Applicable constraints include 2D structure constraints for all models.

1 nt: nucleotide.

2 KB: knowledge-based.

3 TD: thermodynamics.

4 HRP: hydroxyl radical probing.

5 NMR: nuclear magnetic resonance.

6 SAXS: small-angle X-ray scattering.

7 MC: Monte Carlo.

8 SA: simulated annealing.

9 MCMC: Markov chain Monte Carlo.

10 MD: molecular dynamics.

11 REDMD: replica exchange discrete molecular dynamics.

12 REMD: replica exchange molecular dynamics.

13 US: umbrella sampling.

14 REMC: replica exchange Monte Carlo.

15 CG: coarse grained.

16 AA: all atom.

**Graph-based model** (Kim et al., 2014) The model maps a 2D structure to a graph, where a helix is represented by two connected vertices bridging the helix ends, a hairpin loop by a vertex attached to the stem, internal loops by a vertex connecting two helices, and N-way junction loops by a vertex connecting N helices. Like the all-atom force field, the connected vertices form bonds, angles, and torsions. For internal loops, a knowledge-based statistical potential is constructed in terms of the bond angles and torsions. For 3-way and 4-way junctions, RNAJAG program (Laing et al., 2013) predicts topologies and determines the coordinates of the junction vertices. In addition, a radius of gyration-related term is added to determine the global compactness. Monte Carlo/simulated annealing (MC/SA) (Van Laarhoven and Aarts, 1987; Robert and Casella, 2013) methods are used for the 3D graph sampling. The model can predict global helical arrangements compatible with a given secondary structure and give all-atom structures.

**Ernwin** (Kerpedjiev et al., 2015) Ernwin is also a helix-based coarse-grained model which maps helices to reduced cylinders and maps loops to edges attached to a helix or connecting two helices. The relative orientations and positions of two helices are determined by the loops connecting them. The model uses Markov chain Monte Carlo (MCMC) simulation to sample the 3D arrangements of the helices in a force field with five energy terms, including steric clash energy, junction closure energy, radius of gyration-related energy, A-minor energy favoring the long-range interaction in A-minor motif, and loop-loop interaction energy. The last three energy terms are knowledge-based potential of mean force. The model can efficiently predict plausible global shapes of large RNAs. The program is available at http://github.com/pkerpedjiev/ernwin. A server of this program can be found at http://rna.tbi.univie.ac.at/ernwin. RNA sequence and secondary structure are essential inputs to the server and only coarse-grained structures can be retrieved at the present time.

**YUP** (Malhotra et al., 1994; Tan et al., 2006) The model uses one bead to represent a nucleotide located at atom P, and uses harmonic potential functions to describe bond stretching, bond angle bending, and torsion angle twisting. The bond equilibrium values are determined from experiment structures and the force constants are fitted to reflect the uncertainties of the bond lengths, angles, and torsions. The model is used to refine low-resolution structures with additional experimental constraints.

**NAST** (Jonikas et al., 2009b) The NAST model simplifies a nucleotide by using only the C3' atom. Harmonic potentials are applied to the bond lengths and angles, and a three-term cosine function is used for torsions, and the energy parameters are fitted to the statistical potentials derived from three large ribosomal RNAs. Moreover, the repulsive term of the Lennard-Jones potential is applied to avoid steric overlap. Secondary structure and tertiary interaction constraints are included in the energy function. MD simulations are used for conformational sampling. It is computationally efficient to sample the conformational space for large RNAs with the given 2D and 3D constraints. This model, however, cannot *de novo* fold RNAs solely based on sequence information since the base information is missing in the model. All-atom structures can be reconstructed by the supporting software C2A (Jonikas et al., 2009a). The software can be downloaded from https://simtk.org/projects/nast.

**iFoldRNA** (Ding et al., 2008; Sharma et al., 2008; Krokhotin et al., 2015) The iFoldRNA model uses three beads to represent a nucleotide. Two beads are positioned at the center of mass of the phosphate group and the five-atom ring sugar. And the third bead is located at the center of the six-atom ring in the nucleobase. The energy functions include the bonded terms and the non-bonded terms. The bonded terms impose constraints on the bond lengths, angles, and torsions derived from experimental structures. For the non-bonded terms, base pairing interactions, phosphate–phosphate repulsion interactions, hydrophobic interactions (a general attraction between all bases) and nucleotide type-based base stacking interactions are considered in the energy function. The energy parameters for base pairing, stacking and hydrophobic interactions are computed by decomposing the sequence-dependent free energy parameters for individual nearest-neighbor hydrogen-bond model (INN-HB) (Mathews et al., 1999) and thus it can be used to study RNA thermodynamic properties. Moreover, loop entropy is explicitly considered in the model. Replica exchange discrete molecular dynamics (REDMD) simulations starting from a linear structure are utilized for conformational sampling. The model can *de novo* fold small RNAs without secondary structure information including pseudoknots and can fold large RNAs given secondary structure constraints. All-atom 3D structures can be generated. The iFoldRNA v2 server is available at https://dokhlab.med.psu.edu/ifoldrna.

**TOPRNA** (Mustoe et al., 2014) The TOPRNA model uses three beads to describe the phosphate, sugar, and base moieties of a nucleotide. The energy functions follow the form of the standard CHARMM potential (Brooks et al., 2009), including three harmonic potentials for the bond lengths, angles and improper torsions, a cosine form potential for proper torsions, and a Lennard-Jones potential for the Van der Waals interactions. However, electrostatic interactions are ignored in the model. The parameters in the energy functions are fitted to the statistical potentials calculated according to a database of native structures. Replica exchange molecular dynamics (REMD) (Sugita and Okamoto, 1999) simulations are performed for conformational sampling. The link to the force field and the related software is as follows: https://brooks.chem.lsa.umich.edu/index.php?page=toprna&subdir=articles/resources/software.

**IsRNA1** (Zhang and Chen, 2018; Zhang et al., 2021) For the IsRNA1 model, four or five beads are used to represent a nucleotide, including atoms P and C4' for the backbone, two beads for the pyrimidine bases, and three beads for the purine bases. A sum of a harmonic potential and a Gaussian potential is used for the bond length, and angle energy. A cosine form potential is used for the torsional angle energy. A combined function of two bond lengths, two angles, and two torsions is used to characterize the canonical base pairing interactions. A sum of two Morse potentials and two Gaussian potentials is used to account for non-canonical base pairing, base-stacking, base-backbone, and backbone-backbone interactions. Moreover, a repulsive Lennard-Jones potential is used to describe the excluded volume effects.

The parameters in the CG force field are optimized by using the statistical potential in Eq. 2. It is noteworthy that an iterative simulated reference state approach is implemented to parameterize the energy functions, which can account for the correlations between different structural variables in the energy functions. Specifically, energy terms are added to the force field sequentially and the probability distribution in the reference state is updated accordingly. In each step, an energy term E(x) is computed from Eq. 2 and added to the existing CG force field *U*. A key point in the theory is that $p_{\text{ref}}\left(x\right)$ for *x* in Eq. 2 is computed from MD simulations with force field *U*. Such a reference state accounts for the chain connectivity, the excluded volume effects, and all the other interactions considered in *U*. Physically, E(x) represents the interaction potential for *x* in excess of *U*. With the updated energy function U+E(x), the simulated distribution $p_{\text{sim}}\left(x\right)$ of *x* would be equal to the observed result $p_{\text{obs}}\left(x\right)$(Hurst et al., 2021).

In the IsRNA1 approach, REMD simulations are utilized to accelerate conformational sampling. Moreover, the model uses template-based Vfold3D (Zhao et al., 2017) and VfoldLA (Xu et al., 2019) programs to generate the initial structures. Users can also provide the initial structures generated by other models such as RNAcomposer (Popenda et al., 2012) and RNA-MoIP (Yao et al., 2017; Reinharz et al., 2021). Secondary structure is an input for IsRNA1 and energy-minimized all-atom 3D structures can be obtained. The standalone software and the server of the IsRNA1 model for RNA 3D structure prediction can be found at http://rna.physics.missouri.edu/IsRNA/index.html. The input to the server includes RNA sequence, secondary structure and optional initial structures, and the output is the energy-minimized all-atom 3D structures.

**Ren’s model** (Xia et al., 2010, 2013) In this model, a nucleotide is reduced to five beads. Specifically, atoms P and C4' are used to represent the backbone, and three bonded pseudo atoms are used to represent the nucleobase. Three harmonic potentials are implemented to characterize the bond lengths, angles, and torsions. And a Buckingham potential is used to describe the non-bonded interactions. The Debye-Huckel potential is utilized for electrostatic interactions. And a distance- and angle-based energy function is used to describe the hydrogen bonding interaction. The parameters in the potential energies are fitted to the statistical potentials derived from a database of native structures. SA simulations are performed to fold RNAs. Small RNAs of different topologies can be folded into near native structures and large RNAs can be folded with experimental constraints. Atomic 3D structures are obtained in the output. The program of this model is not released.

**oxRNA** (Šulc et al., 2014) In the oxRNA model, a nucleotide is treated as a rigid body with five interaction sites including backbone, hydrogen-bonding, cross-stacking and 3′- and 5′-stacking interactions. The energy functions adopt relatively complex forms and the energy parameters are fitted to reproduce the thermodynamics properties of the nearest-neighbor model (Tinoco et al., 1973) for short duplex and hairpin structures. The virtual move Monte Carlo (VCMC) algorithm combined with umbrella sampling (US) method or MD simulations are used for conformational sampling. It not only can fold small RNAs including pseudoknot and kissing hairpin structures, but also can characterize the thermodynamic and mechanical properties of RNAs. The model can be downloaded from https://dna.physics.ox.ac.uk/index.php/Main_Page. The web server (Poppleton et al., 2021) was recently released at https://oxdna.org/.

**SimRNA** (Boniecki et al., 2016; Magnus et al., 2016) The SimRNA model reduces 20–23 heavy atoms down to five, namely atoms P, C4', C2, N1 and C4 for pyrimidines, N9 and C6 for purines. The energy function in SimRNA model consists of sequence-independent local bonded terms and sequence-dependent non-bonded terms. The non-bonded terms include base-base, base-backbone, and backbone-backbone interactions, and discrete statistical potentials are constructed for these interactions based on a database of native structures. Replica exchange Monte Carlo (REMC) simulations are used to sample the conformational space. The model can fold small RNAs without secondary structure constraint and can fold large RNAs with secondary structure and other interatomic distance constraints. The standalone software can be found at http://genesilico.pl/software/stand-alone/simrna. The server of the model can be accessed from https://genesilico.pl/SimRNAweb. Users feed RNA sequence and 2D structure (optional) to the server, and can fetch the predicted all-atom 3D structures after the jobs are done.

**SPQR** (Poblete et al., 2018) The SPQR model uses a bead as the phosphate group, three beads for the nucleobase forming a rigid triplet, and a virtual bead for the sugar attached to the base. The non-bonded energy functions account for excluded volume effects, base pairing, base stacking, base-phosphate, and backbone-backbone interactions. The potentials of mean force are applied to the non-bonded interactions and the relative strengths of different interactions are trained by distinguishing native structures from decoys for dozens of internal and hairpin loop structures. SA/MC protocol is adopted for sampling. All-atom structures are reconstructed finally. This model can provide accurate predictions for the secondary and tertiary structures of small RNA motifs. The program can be downloaded at http://github.com/srnas/spqr.

**HiRE-RNA** (Pasquali and Derreumaux, 2010; Cragnolini et al., 2013; Sterpone et al., 2014; Cragnolini et al., 2015) In the HiRE-RNA model, the coarse-grained representation of a nucleotide consists of six or seven beads: five beads for the backbone (atoms P, O5', C5', C4', C1'), one bead (for pyrimidine) or two beads (for purine) placed at the centers of mass of heavy atoms in the all-atom rings in the base. The energy functions include the local bonded terms and the long-range non-bonded terms. For the local energy terms, harmonic potentials are employed to constrain bond lengths and angles, and the cosine form potential is applied to torsions. For the non-local energy terms, excluded volume potential, electrostatic potential, base stacking potential, and base-pairing potential including non-canonical base pairs are considered. The key parameters in the non-bonded energies are the relative strengths between different energy terms. The parameters are trained using the genetic algorithm to distinguish native structures from decoys. REMD simulations are performed for conformational sampling. The model can *de novo* fold small RNAs of different topologies from fully extended structures without using secondary structure constraints, and can fold large RNAs with secondary structure and additional experimental constraints.

Besides 2D structure constraints, some of the models above can integrate additional constraints into energy functions to make conformational sampling more efficient and effective for structure prediction. The YUP model (Malhotra et al., 1994; Tan et al., 2006) can use footprinting data (Tijerina et al., 2007; Sclavi et al., 1998) and cross-linking (Harris and Christian, 2009) experimental information to refine structures. The iFoldRNA model (Ding et al., 2008; Sharma et al., 2008; Krokhotin et al., 2015) can utilize the hydroxyl radical probing (HRP) (Ding et al., 2012) and NMR experimental data. Ren’s model (Xia et al., 2013) can transform NMR and small-angle X-ray scattering (SAXS) (Lipfert and Doniach, 2007) experimental data to constrain the sampling space. The NAST (Jonikas et al., 2009b) and SimRNA (Boniecki et al., 2016; Magnus et al., 2016) models can implement interatomic distance constraints when performing simulations.

The CG models reviewed above can be classified into two categories, namely the helix-based and the nucleotide-based models. The helix-based models can predict the global structures efficiently especially for large RNAs, but sequence information and structure details are missing in the sampling process and secondary structures are essential as input. The Graph-based model performs junction prediction first with RNAJAG program (Laing et al., 2013), which can reduce the conformational sampling space for RNAs with junction structures but may incur the risk of using an nonnative junction topology. The helix-based model Ernwin implements new potentials favoring A-minor motif and loop-loop interactions but the atomic 3D structures are not available at the present time.

The nucleotide-based models can capture structural details at the expense of computational cost. The low-resolution models YUP and NAST using one bead per nucleotide have the advantage over high resolution models in terms of computational speed but they cannot accurately model the different (non-)canonical base pairing interactions which are important in stabilizing RNA tertiary structures. Like the helix-based models, YUP and NAST models cannot fold an RNA solely based on sequence information and additional 2D and 3D restraints are required. The high resolution models using 3-7 beads per nucleotide introduce a force field consisting of the bonded and non-bonded energy terms and the differences between models lie in the energy components and energy function forms.

For the bonded energy terms, harmonic potentials or Gaussian potentials or combination of both are often used to restrain the bonds and angles, and a cosine form function is used for the torsions in the MD-based models TOPRNA, IsRNA1, Ren’s model, and HiRE-RNA, while discrete statistical potentials are used for the bonds, angles, and torsions in the MC-based models SimRNA and SPQR.

For the non-bonded energy terms, excluded volume potential, electrostatic repulsive energy between phosphate-phosphate groups, and backbone-backbone, base-backbone, and base-base interactions are the main energy components. Although different models may consider different interactions, the base-base energies are included in all models because base pairing and base stacking interactions are the most important forces stabilizing RNA structures. The different models adopt different strategies to account for base-base energies. For the canonical base pairing interactions, all the models use explicit terms of various energy functions to favor the canonical base pairs, and in these energy terms, distances, angles and torsions are introduced to characterize the co-planarity and the relative positions and orientations of two paired bases. For the non-canonical base pairing and base stacking interactions, several models use explicit terms to describe the interaction potential energy. For example, the iFoldRNA model uses a general base attraction energy to mimic the non-canonical base pairing interactions and uses distance-based energy functions to favor base stacking interactions. The oxRNA model does not consider non-canonical base pairing interactions, but uses complex distance- and angle-dependent energy functions to describe the stacking interactions, and the energy parameters are fitted by reproducing the melting temperatures. The SPQR model uses the distance-, angle-, and torsion-based statistical potentials to describe the different non-canonical base pairing and base stacking interactions. The SimRNA model implements statistical potentials to extract the energy functions using six discrete distances as the variables. The HiRE-RNA model uses functions of distances and angles to achieve base pairing and stacking interactions and the energy parameters are determined by using the genetic algorithm to distinguish native structures from decoys. In contrast, other models use implicit energy terms to describe non-canonical base pairing and base stacking interactions. For example, the IsRNA1 model and the Ren’s model integrate the non-canonical base pairing, base stacking, base-backbone and backbone-backbone interactions into an effective potential as a function of the distances between non-bonded atom pairs such as the Van der Waals energy in the all-atom force field. The IsRNA1 model uses a sum of a Morse potential and two Gaussian potentials while Ren’s model uses a Buckingham potential, and the corresponding energy parameters are fitted to the interatomic distance statistical potentials. The non-canonical base pairing interactions are of great importance but the current models either ignore them or describe them with low accuracy. The statistical potential-based energy for the non-canonical base pairs could underestimate the interaction strength of relatively rare base pairs in the native structures. The implicit and CG potentials for non-canonical base pairs have the difficulty in accurate description of the geometries and the interaction strength.

It is important to note that other excellent CG RNA modeling methods have been reported elsewhere but cannot be covered with great detail in this short mini-review. For example, NARES-2P (He et al., 2013), a physics-based 2-bead model, can fold DNA and RNA double helical structures, featuring the mean-field dipole-dipole interactions between bases. Combined with global optimization conformational space annealing algorithm and limited distance restraints, NARES-2P model can also deal with complex RNA folds (Sieradzan et al., 2018). RECER, a 5-bead CG model, can accurately predict the native structures and capture the folding free energy of RNA hairpins and duplexes (Bell et al., 2017). Fyta et al. has developed a four-bead CG model for double-stranded RNA which uses density functional theory to determine the parameters in energy functions and can reproduce the structural and mechanical properties (Cruz-León et al., 2018). Tan et al. managed to predict the 3D structures and stability of RNA kissing complexes in monovalent/divalent ion solutions by using a 3-bead CG model (Jin et al., 2019). Even for the large ribosome system, a 1-bead CG model for RNAs and proteins has been applied to study its dynamics (Trylska et al., 2005). Recently a three-bead CG model has been used to study the folding of the ribosomal subunit where the $Mg^{\mathrm{2+}}$ effect can be explicitly considered (Hori et al., 2021).

### 2.2 Performance Comparison Between the iFoldRNA, SimRNA and IsRNA1 Models

Three CG methods, namely iFoldRNA, SimRNA, and IsRNA1 models, are compared in terms of performance for RNA 3D structure prediction in the reference paper (Zhang et al., 2021). The other methods are not chosen here because the programs are not open to the public, or generate only CG structures, or are designed mainly for studying the folding thermodynamics of small RNAs instead of structure prediction of large RNAs. A large dataset of 130 RNAs are used for benchmark test. The dataset includes 44 stem-loops, 43 multi-way junctions, and 43 structures of long-range tertiary interactions, and the shortest and longest RNAs contain 40 and 161 nucleotides, respectively. RNA sequences and native secondary structures are used as inputs to the three models. The IsRNA1 model uses template-based Vfold3D (Cao and Chen, 2011; Xu and Chen, 2015; Zhao et al., 2017) and loop-based VfoldLA (Xu and Chen, 2017; Xu et al., 2019) algorithms to generate initial 3D structures. As an objective test, the native 3D structures in the benchmark dataset and their homologous entries are excluded from the template/loop database when generating initial structures. For the 130 RNAs, the average/median RMSDs for the predicted top-ranked structure by IsRNA1, SimRNA, and iFoldRNA models are 9.51/8.12, 11.26/10.95, and 11.87/11.37 Å, respectively. Considering the top-three predicted structures, the average RMSDs for the best model predicted by IsRNA1, SimRNA, and iFoldRNA models are 8.34, 9.73, and 10.88 Å, respectively. For all the three models, however, the average RMSDs for multi-way junction structures are always larger than those for structures of other topologies, which indicates that the relative positions of the helices in the junction structures are more difficult to predict. For RNAs larger than 100 nucleotides, the RMSDs for the three models are almost larger than 20 Å and the average clash score (the number of serious all-atom steric overlaps per 1,000 atoms) (Word et al., 1999) for the IsRNA1 model (4.0) is lower than that for the SimRNA model (139.7) and the iFoldRNA model (170.4) as energy minimization is performed for the predicted structures in IsRNA1 model. Detailed results can be found in the reference paper (Zhang et al., 2021).

The runtime for 3D structure prediction is dependent on RNA size. For an RNA of about 100 nucleotides, the runtimes for IsRNA1 (50 million steps), SimRNA (16 million steps), and iFoldRNA (2 million steps) models are about 1 day, 1 day, and 15 h, respectively. For an RNA of about 150 nucleotides, the runtimes increase to about 2 days, 3.5 days, and 20 h. All the three models provide server services to users, and users can adjust and test the simulation time or step based on the previous runtime estimation.

The performance improvement for the IsRNA1 model may result from the following aspects: 1) a set of more detailed energy functions are used for the bonds, angles, and torsions instead of the simple harmonic potentials in the previous models, enabling broader and more accurate conformational coverage for RNA backbone; 2) the iterative simulated reference state approach takes into consideration the correlation between different energy terms to avoid over-constraint; 3) the iteratively constructed energy functions learn knowledge not only from the native structures but also from the non-native ones in the simulated reference state; 4) using the structures generated by the template-based methods Vfold3D and VfoldLA as the initial structures may help folding from an initial state not too far away from the native structures. Meanwhile, the relatively poor performance for the junction structures of the CG models including the IsRNA1 model necessitates a more detailed energy function, especially for non-canonical base pairing and stacking interactions, for this structural topology.

## 3 Future Developments in Modeling Ribonucleic acid 3D Structures

Compared with all-atom models, CG models have a smoother energy landscape and far fewer atoms (pseudo-atoms), and thus the conformational space can be explored more efficiently. To further speed up the computation, GPU-driven CG models could be a promising option justified by the GPU version of all-atom MD simulation software AMBER. General MD simulation engines LAMMPS (Plimpton, 1995) and OpenMM (Eastman et al., 2017) have already supported GPU computation, enabling new CG models on these platforms.

Even with native secondary structures, the accuracy of structure prediction for large RNAs (>100nt) is low. Large RNAs often contain multi-way junctions, and an incorrect arrangement of the helices in the junction structures could result in large deviation from native structures. The problem can be caused by inaccurate energy functions or insufficient sampling or both. Insufficient sampling problem could be alleviated by extending simulation time or adopting advanced accelerating algorithms. However, inaccurate energy problem is more serious because energy functions are used not only to assess structures but also to bias the sampling direction in the conformational space. Inaccuracy in energy functions may come from the inaccurate characterization of both the intra-RNA interactions and RNA-environment interactions. The RNA-environment interactions, including RNA-solvent, RNA-ion, RNA-ligand and RNA-protein interactions, are missing or not accurate in the CG models. Instead of *de novo* folding, the combination of CG models and homology modeling of whole structures or motifs has the potential to improve the performance as in the IsRNA1 model.

Solving structures by traditional X-ray crystallography and NMR spectroscopy experiments is laborious and technically challenging. However, low-resolution information (overall shape, tertiary contact, local properties, etc.) may be readily available from experiments such as SAXS (Small-angle X-ray scattering) (Lipfert and Doniach, 2007), cryo-EM (Electron microscopy) (Bai et al., 2015), FRET (Förster resonance energy transfer) (Jares-Erijman and Jovin, 2003; Stephenson et al., 2016), chemical cross-linking (Harris and Christian, 2009), footprinting (Tijerina et al., 2007; Sclavi et al., 1998). CG models combined with the low-resolution information can significantly improve the structure prediction results (Ponce-Salvatierra et al., 2019; Li et al., 2020). Efficient and accurate integration of experimental data and CG models is a highly promising approach for the prediction of large RNA structures.
